# Analysis of anaesthesia services to calculate national need and supply of anaesthetics in Switzerland during the COVID-19 pandemic

**DOI:** 10.1371/journal.pone.0248997

**Published:** 2021-03-19

**Authors:** Christoph Karl Hofer, Pedro David Wendel Garcia, Christof Heim, Michael Thomas Ganter

**Affiliations:** 1 Anaesthesiology, Schulthess Clinic, Zurich, Switzerland; 2 Swiss Society for Anaesthesiology and Resuscitation (SSAR), Committee on Data and Quality Management, Berne, Switzerland; 3 Institute of Anaesthesiology, Kantonsspital Winterthur, Winterthur, Switzerland; University of Bern, University Hospital Bern, SWITZERLAND

## Abstract

**Background:**

In Switzerland, details of current anaesthesia practice are unknown. However, they are urgently needed to manage anaesthesia drug supply in times of drug shortages due to the pandemic.

**Methods:**

We surveyed all Swiss anaesthesia institutions in April 2020 to determine their annual anaesthesia activity. Together with a detailed analysis on anaesthetic drug use of a large, representative Swiss anaesthesia index institution, calculations and projections for the annual need of anaesthetics in Switzerland were made. Only those drugs have been analysed that are either being used very frequently or that have been classified critical with regard to their supply by the pharmacy of the index institution or the Swiss Federal Office of Public Health.

**Results:**

The response rate to our questionnaire was 98%. Out of the present 188 Swiss anaesthesia institutions, 185 responded. In Switzerland, the annual number of anaesthesias was 1’071’054 (12’445 per 100’000 inhabitants) with a mean anaesthesia time of 2.03 hours. Teaching hospitals (n = 54) performed more than half (n = 572’231) and non-teaching hospitals (n = 103) provided almost half of all anaesthesias (n = 412’531). Thereby, private hospitals conducted a total of 290’690 anaesthesias. Finally, office-based anaesthesia institutions with mainly outpatients (n = 31) administered 86’292 anaesthesias. Regarding type of anaesthesia provided, two thirds were general anaesthesias (42% total intravenous, 17% inhalation, 8% combined), 20% regional and 12% monitored anaesthesia care. Projecting for example the annual need for propofol in anaesthesia, Switzerland requires 48’573 L of propofol 1% which corresponds to 5’644 L propofol 1% per 100’000 inhabitants every year.

**Conclusions:**

To actively manage anaesthesia drug supply in the context of the current pandemic, it is mandatory to have a detailed understanding of the number and types of anaesthesias provided. On this basis, the Swiss annual consumption of anaesthetics could be projected and the replenishment organized.

## Introduction

The COVID-19 pandemic poses unprecedented, worldwide challenges for all nations. Barriers to accessing medicines are nothing new, but the COVID-19 pandemic endangers the stability and integrity of pharmaceutical supply chains. Anaesthesia services and critical care medicine are the two main stakeholders using identical drugs for sedation, mechanical ventilation including paralysis and pain relief. The rapid spread of the infection has led to a surge in the rate of hospitalizations and ICU admissions creating pressure on the supply chain resulting in shortages in these anaesthesia and critical care medications [1–3].

During the first wave of the pandemic in Switzerland, the Swiss Federal Office of Public Health (BAG) prohibited all non-urgent elective surgical cases over a 6 week period in March and April 2020 [4]. Thereby, resources like staff, mainly specialized nurses and physicians, materials and drugs were shifted from non-COVID-19 to COVID-19 units in order to prevent a bottleneck in these resources–a reaction that happened in most countries [5]. In the second wave starting end of October 2020 however, no federal restrictions on surgical and anaesthesia activity were passed by law. It was expected that both, COVID-19 and non-COVID-19 units run in parallel as far as possible and that drug shortage would neither impact one or the other unit. In order to plan for national anaesthesia drug demands and its supply, the prerequisite was that we had an estimate of the annual number and types of anaesthesia performed in Switzerland.

Until now, exact data on anaesthesia services in Switzerland are lacking. Indeed, the Swiss Society for Anaesthesiology and Resuscitation (SSAR) runs a national quality improvement programme A-QUA _CH_ (Anaesthesia QUAlity in Switzerland, A-QUA) since 2014. However, only 103 out of 188 Swiss anaesthesia departments have participated in the first part of the A-QUA (data on institutions, structure) so far, and the second part of the programme (data from individual anaesthesia cases, process and outcome) was just launched in 2017 (https://sgar-ssar.ch/a-qua/).

Since not all Swiss anaesthesia departments have participated in A-QUA yet, the *first aim* of our study was to perform a survey in all Swiss anaesthesia departments on numbers, times and type of anaesthesia provided in the previous year (2019). The *second aim* was to calculate an estimate of the annual need for anaesthetics, analgesics and other drugs used in anaesthesia under normal conditions in Switzerland. Because no national data are available, data from a large, representative index anaesthesia institution in Switzerland was analysed and served as basis for further calculations. In combination with the survey, this allowed us to make a projection of the Swiss annual need and consumption of selected and critical drugs in anaesthesia.

## Materials and methods

This retrospective observational questionnaire study was conducted upon request of the Swiss Federal Office of Public Health (BAG) in April 2020 to coordinate the demand and supply of anaesthetics for our national anaesthesia activity in the context of the emerging COVID-19 pandemic. STROBE recommendations for observational studies were followed [6].

### Swiss definition of anaesthesia

Anaesthesia activity is defined as any surgical, interventional or diagnostic procedure where a dedicated anaesthesia team (physician and nurse anaesthesia provider) is responsible for patient care. It includes neuraxial blocks performed for labour pain relief and assisted delivery as well as anaesthesia care for life-threatened patients including resuscitation, management in the emergency department (ED) and in-hospital transport to a site of procedure (e.g. CT scan or operating theatre). In Switzerland, it does not include sedation and analgesia delivered by non-anaesthetists (including ICU management, routine endoscopies etc.) or specialist interventional pain procedures.

According to the definitions in the Swiss anaesthesia quality improvement programme A-QUA, each anaesthesia is classified into one of five categories: general anaesthesia by the total intravenous route (GA, TIVA), general anaesthesia by inhalation (GA, inhalation), regional anaesthesia (RA), combined GA & RA, and monitored anaesthesia care (MAC; includes anaesthesia standby and sedation).

### Swiss anaesthesia landscape

Switzerland has a population of 8’606’033 people (end of 2019), consists of 26 member states (cantons) and has three main language regions, which have significant impact on culture and social life. Roughly, around two third speak German (German part, including the very small Romansh part, since most of these people also speak German), a quarter speaks French, and less than 10% speak Italian (https://www.bfs.admin.ch/bfs/en/home.html). According to the Swiss anaesthesia quality improvement programme A-QUA, there are currently 188 Swiss anaesthesia institutions. Anaesthesia is basically performed in three different settings:

- Teaching hospitals with residency programs for anaesthesiology (n = 54). Level A1 training centres (n = 10) and A2 training centres (n = 13) typically includes the large Swiss hospitals, i.e. university hospitals and cantonal hospitals, where a large number of anaesthesias (A1 ≥12’500 and A2 ≥7’500 anaesthesias annually) is performed with a high degree of complexity. In the level B training centres (n = 22) there are medium-sized hospitals (annual number of anaesthesias ≥3’500) and in the level C training centres (n = 9) there are small regional hospitals (annual number of anaesthesias ≥1’000).
- Non-teaching hospitals (n = 103). Hospitals that do not participate in the specialist medical training for anaesthesiology. They are of different legal forms and sponsorship (public, private), sizes and perform anaesthesia of varying complexity. Examples include smaller public hospitals (regional hospitals, n = 41) and the heterogeneous group of private hospitals (n = 62).
- Office-based anaesthesia institutions, mainly outpatients (n = 31). This group includes both large, mainly outpatient companies, but also small outpatient anaesthesia sole traders.

### Participants and questionnaire

All 188 Swiss anaesthesia institutions were contacted by e-mail and phone to participate in the study. After the initial round, we reminded them by phone twice if the questionnaire was not returned in due time. Agreement to respond was considered as consent to participate. The questionnaire was based on 7 questions concerning number, duration and type of anaesthesia at each institution (S1 File). Anaesthesia time has been defined according to the joint German, Austrian and Swiss recommendation on perioperative procedural times (K14b, start to end of anaesthesia care) [7].

### Annual need of drugs at index anaesthesia institution

Detailed data on the annual drug use overall and for every single type of anaesthesia were available to the authors from a large, representative index anaesthesia institution in Switzerland (Institute of Anaesthesiology, Kantonsspital Winterthur, Switzerland; annual number of anaesthesias around 18’000). We based our calculations on drugs ordered and delivered to the index institution in 2019. Thereby, the calculations do not directly reflect the actual quantity administered per patient. In fact, it represents the average quantity administered per patient including all discarded quantities of medication, leftovers and further losses (e.g., remaining drugs in syringes, broken vials, ampoules that have been expired).

### Swiss annual need of anaesthetics, projection

Data on annual drug use were taken from the index anaesthesia institution to make projections for the total Swiss annual need for medication in anaesthesia. Only those drugs have been analysed that are either being used very frequently or that have been classified critical with regard to their supply by the pharmacy of the index institution or the Swiss Federal Office of Public Health. We only included drugs being used during anaesthesia. Thereby, no calculations were made for pain therapy following anaesthesia in the first days after surgery.

To calculate the Swiss annual need, the following assumptions form the basis of the projections:

- Correction for the length and type of the anaesthesia. The mean length and type of anaesthesia varies between institutions. Accordingly, the differences in length and types between index and average Swiss anaesthesia institution was considered in the projections.
- GA, inhalation. This type of anaesthesia is mostly started with an IV induction by propofol. Thiopental or etomidate are being used alternatively. However, since these drugs are rarely being used, only propofol has been taken into account in the current calculations. Regarding inhaled anaesthesia drugs, sevoflurane is predominantly being used in Switzerland. We projected that 95% is done by sevoflurane and 5% by desflurane.
- Muscle relaxation and its reversal. Rocuronium is the most commonly used muscle relaxant. Alternatives like succinylcholine, atracurium, cisatracurium or mivacurium are rarely being used. In roughly two thirds of GA with endotracheal intubation, muscle relaxation needs to be reversed at the end. Here we assumed that reversal is predominantly done with neostigmine (three quarters) and less frequently by sugammadex (one quarter).
- Local anaesthetics. We did the calculation only for the most relevant drugs, so that local anaesthetics for spinal, epidural and peripheral RA (alone or combined with GA) are covered. The medication for pain service was not included.
- Anaesthetics used for one MAC were assumed to be 200 mg of propofol and 0.1 mg of remifentanil (i.e., 1 mg in every tenth patients).

### Statistical analysis

Due to the observational character of this study no prespecified power calculation was needed. Calculations and substratifications per group of the Swiss annual anaesthetic consumptions were approached by extrapolation of the annual anaesthetic consumption at the index anaesthesia institution corrected for both, the total number of anaesthesias and the average time of anaesthesia per centre and per anaesthesia type. Comparisons of population characteristics were performed using the Wilcoxon Signed Rank and chi-squared test, as appropriate. Correlations were calculated by means of linear regression fitting, *R-squared* values were provided to quantify the absolute degree of correlation. A two-sided *p* <0.05 was considered statistically significant. Statistical analysis was performed via a fully scripted data management pathway using the R environment for statistical computing version 3.6.1 [8]. Values are given as medians with interquartile ranges or counts and percentages as appropriate.

## Results

**Fig 1:**
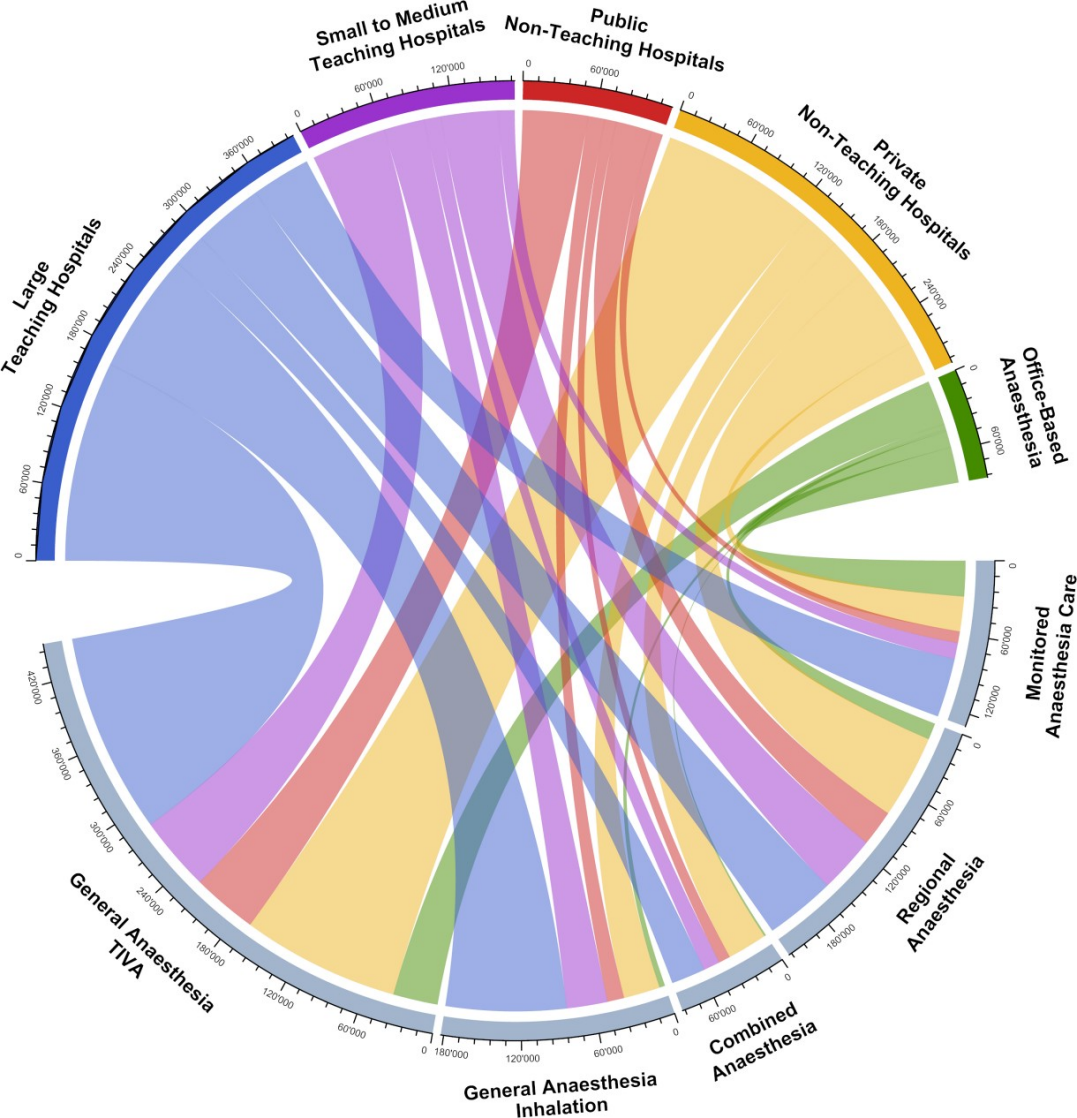
Swiss anaesthesia institutions and types of anaesthesia. Each anaesthesia service is classified into one of five categories in Switzerland (lower part of the figure): general anaesthesia by the total intravenous route (General Anaesthesia, TIVA), general anaesthesia by inhalation (General Anaesthesia, inhalation), regional anaesthesia (RA), GA & RA (Combined Anaesthesia), and monitored anaesthesia care (MAC; includes anaesthesia standby and sedation). These types of anaesthesias are done at the following institutions (upper part of the figure): Large teaching hospitals (n = 23) 41%, 26%, 14%, 7%, and 12% respectively; small to medium teaching hospitals (n = 31) in 36%, 20%, 28%, 8%, and 8% respectively; public non-teaching hospitals (n = 41) in 46%, 12%, 25%, 9%, and 8% respectively; private non-teaching hospitals (n = 62) in 45%, 10%, 24%, 11%, and 10% respectively; office-based anaesthesia (n = 31) 43%, 5%, 16%, 2%, and 34% respectively. The numbers on the circle correspond to the number of anaesthesias.

The response rate to our questionnaire was 98%. Out of the present 188 Swiss anaesthesia institutions, 185 responded. Table 1 provides a summary of all anaesthesia institutions and activities in 2019. Overall, 1’071’054 anaesthesia procedures (12’445 per 100’000 inhabitants) with a mean anaesthesia time of 2.03 hours were performed in Switzerland. Teaching hospitals with a residency program for anaesthesiology (n = 54) perform more than half of the anaesthesias (n = 572’231), their average duration of anaesthesia being 2.30 hours. In contrast, non-teaching hospitals (n = 103) provide almost half of all anaesthesia services (n = 412’531) with an average duration of anaesthesia of 1.84 hours. Thereby, private hospitals (n = 62) conduct a total of 290’690 anaesthesias. Finally, office-based anaesthesia institutions with mainly outpatients (n = 31) administer 86’292 anaesthesias with an average duration of anaesthesia 1.08 hours. Regarding the type of anaesthesia provided, two thirds were GA (42% TIVA, 17% inhalation, 8% combined), 20% RA and 12% MAC (Fig 1).

**Table 1:** Anesthesia institutions and services in Switzerland in 2019.

	Switzerland overall	German	French	Italian	*P*
**Inhabitants**, n (%)	**8’606’033**	6’027’928 (70)	2’226’614 (26)	351’491 (4)	
**Anesthesia institutions**, n (%)	**188**	140 (74)	39 (21)	9 (5)	
*Teaching hospitals*	***54***	*40 (74)*	*11 (20)*	*3 (6)*	
*Non-Teaching hospitals*	***103***	*72 (70)*	*25 (24)*	*6 (6)*	
*Office-based anesthesia institutions*	***31***	*28 (90)*	*3 (10)*	*0 (0)*	
**Number of anesthesias**, n (%)	**1’071’054**	752’765 (70)	268’277 (25)	50’012 (5)	
*per 100’000 inhabitants*	***12’445***	*12’488*	*12’049*	*14’229*	*<0*.*001*
**Anesthesia time**, hours (%)	**2’168’889**	1’553’348 (72)	518’327 (24)	97’214 (4)	
*per 100’000 inhabitants*	***25’202***	*25’769*	*23’279*	*27’658*	*<0*.*001*
*Mean anesthesia time*	***2*.*03***	*2*.*06*	*1*.*93*	*1*.*94*	*<0*.*001*
**Type of anesthesia**, %					
*GA*, *TIVA*	***42***	*48*	*27*	*28*	*<0*.*001*
*GA*, *inhalation*	***17***	*14*	*26*	*19*	*<0*.*001*
*RA*	***20***	*19*	*23*	*23*	*<0*.*001*
*Combined GA & RA*	***8***	*7*	*9*	*12*	*<0*.*001*
*MAC*	***12***	*11*	*14*	*17*	*<0*.*001*

Switzerland has three main language regions, which have significant impact on culture and social life. Roughly, around two-third speaks German (German part, including the very small Romansh part), a quarter speaks French, and less than 10% speaks Italian.

GA = general anaesthesia; GA, TIVA = GA by total intravenous route; MAC = monitored anaesthesia care; RA = regional anaesthesia.

Looking at the three main language regions in Switzerland, we could find significant differences in the distribution of anaesthesia institutions, number of anaesthesias per 100’000 inhabitants and the type of anaesthesia (Table 1). With 70% inhabitants, the German part of Switzerland is the largest part of the country, provides 70% of all anaesthesia services and hosts 74% of all Swiss anaesthesia institutions. It is noticeable that GA is administered more often intravenously (TIVA) and less often by inhalation compared to the rest of Switzerland. Furthermore, 90% of all office-based anaesthesia institutions are located in the German part. The number of anaesthesias per 100’000 inhabitants is significantly lower in the French part (12’049 per 100’000) and higher in the Italian part (14’229 per 100’000). Looking at the type of anaesthesia, both the French and the Italian part use significantly more inhaled anaesthetics and provide more RA and MAC compared to the German part.

The average use of drugs per one anaesthesia delivered at our index anaesthesia institution is shown in S1 Table. Only those drugs have been listed which have a high turnover or which have been classified critical, especially with regard to their supply chain. There were some differences between the index anaesthesia institution and the average Swiss anaesthesia institution. It must be noted that the mean duration of anaesthesia was longer at the index institution (2.49 hours) compared to 2.03 hours (mean duration in all Swiss anaesthesia institutions). In addition, there were some differences in the distribution of the type of anaesthesia (index institution vs. all Swiss anaesthesia institutions): GA, TIVA (52 vs. 42%), GA, inhalation (23 vs. 17%), RA (17 vs 20%), combined GA & RA (2 vs. 8%), MAC (3 vs 12%). These differences have been taken into account in the calculation and projection of the Swiss annual need of selected and critical drugs in anaesthesia.

Table 2 shows the projections of the annual need of selected drugs for Swiss anaesthesia services, corrected for type and duration of anaesthesia. Looking at propofol for example, Switzerland requires 48’573 L of propofol 1% annually (485.7 kg pure substance), which equals 2’428’636 vials at 20 mL. Thereby, yearly 5’644 L propofol 1% are required for anaesthesia services per 100’000 Swiss inhabitants, and 45’350’392 mg propofol are required per 100’000 anaesthesias in Switzerland.

**Table 2:** Estimated annual need of selected drugs for Swiss anesthesia services.

Selected and most relevant drugs	Swiss annual need, overall	*per 100*,*000 inhabitants*	*per 100*,*000 anesthesias*
**Hypnotics**			
Propofol (mg)	485’727’189	5’644’031	45’350’392
Volatile anesthetic drug (mL)	7’098’450	82’482	662’754
• Sevoflurane (mL)	6’530’574	75’884	609’733
• Desflurane (mL)	567’876	6’599	53’020
**Opioids**			
Fentanyl (mg)	362’079	*4’207*	*33’806*
Remifentanil (mg)	301’732	*3’506*	*28’172*
Morphine (mg)	3’620’788	*42’073*	*338’058*
**Muscle relaxants**			
Rocuronium (mg)	32’587’092	*378’654*	*3’042’526*
Reversal of muscle relaxants			
• Glycopyrrolate 0.5 mg / Neostigmine 2.5 mg (vials)			
	244’205	*2’838*	*22’800*
• Sugammadex (mg)	4’453’145	*51’744*	*415’772*
**Anti-emetics**			
Dexamethasone (mg)	1’723’798	*20’030*	*160’944*
Ondansetron (mg)	1’077’373	*12’519*	*100’590*
**Local anesthetics**			
Bupivacaine (mg)	1’722’419	*20’014*	*160’815*
Lidocaine (mg)	53’247’459	*618’722*	*4’971’501*
Mepivacaine (mg)	38’275’598	*444’753*	*3’573’638*
Ropivacaine (mg)	11’722’019	*136’207*	*1’094’438*
**Cardiovascular drugs**			
Ephedrine (mg)	24’774’191	*287’870*	*2’313’066*
Norepinephrine (mg)	348’387	*4’048*	*32’527*
Phenylephrine (mg)	30’968	*360*	*2’891*
Atropine (mg)	65’551	*762*	*6’120*
Epinephrine (mg)	30’968	*360*	*2’891*
Clonidine (mg)	15’483	*180*	*1’446*
**Others**			
Crystalloids (LR, RF, NaCl 0.9%; L)	1’056’775	*12’279*	*98’667*
Carrier solutions (NaCl 0.9%, D5W; L)	165’315	*1’921*	*15’435*
Midazolam (mg)	232’258	*2’699*	*21’685*
Ketamine (mg)	4’645’161	*53’976*	*433’700*

Crystalloids, carrier solutions (short infusions): NaCl 0.9% = normal saline, LR = lactated Ringer’s solution, D5W = dextrose 5% in water, RF = ringerfundin.

Bupivacain = Bupivacain 0.5% hyperbaric for spinal anaesthesia; Lidocaine = Lidocaine 2% for test dose and top up of epidural anaesthesia; Mepivacaine = Mepivacaine 1% for peripheral nerve block; Ropivacaine = Ropivacaine 0.2% for running epidural anaesthesia and Ropivacaine 0.5% for peripheral nerve block.

**Fig 2:**
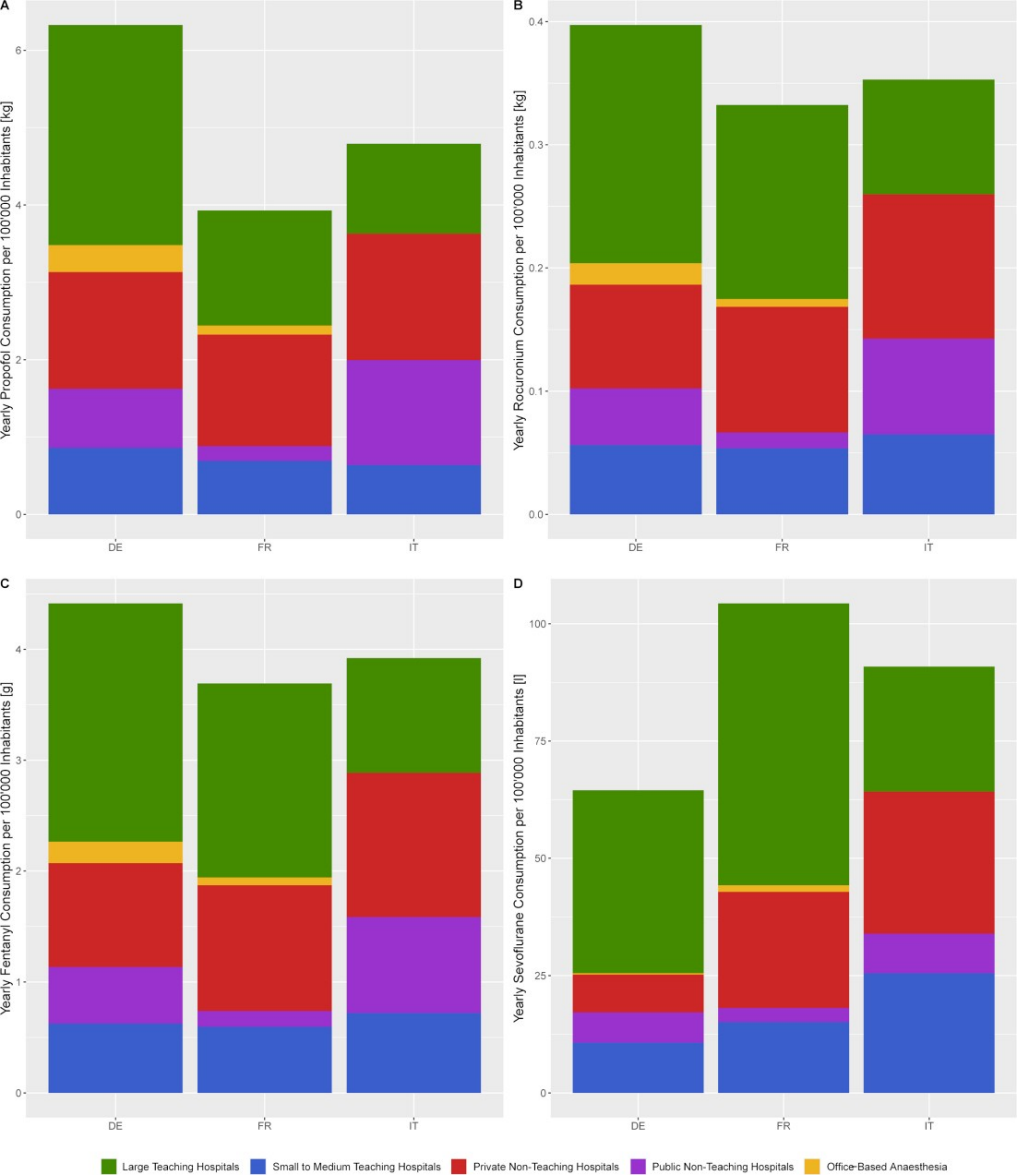
Swiss annual need of anaesthetics per language region and type of anaesthesia institution. The annual cumulated consumption is exemplified for the following anaesthetics: A) Propofol, B) Rocuronium, C) Fentanyl, D) Sevoflurane. The stacked sub-plots for each anaesthetic present the stratification by language region (bar) into DE–German region, FR–French region and IT–Italian region, and by the type of anaesthesia institution (bar subdivision) into large teaching hospital (green), small to medium teaching hospital (blue), private non-teaching hospital (red), public non-teaching hospital (purple) and office-based anaesthesia (yellow).

**Fig 3:**
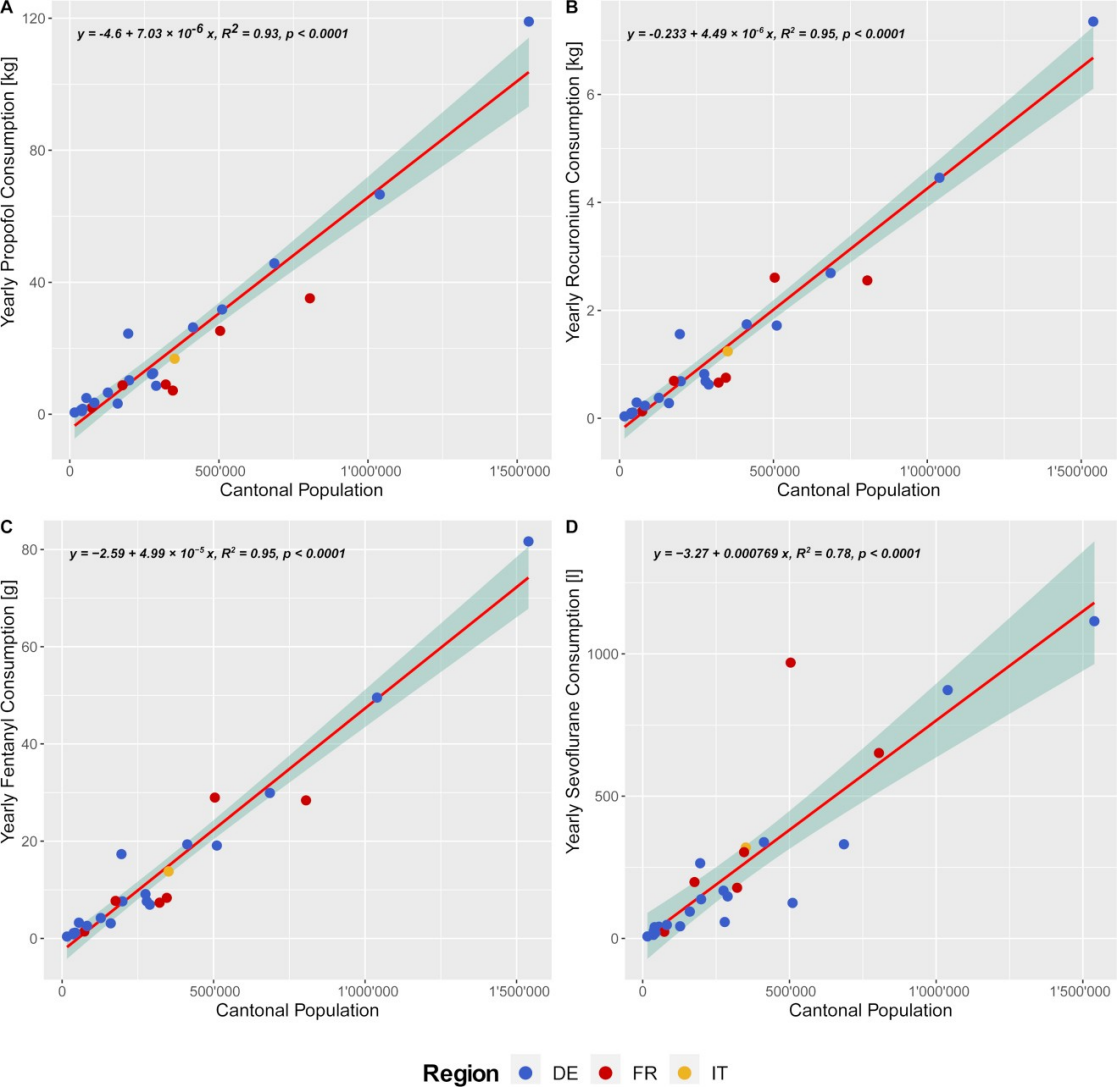
Swiss annual anaesthetic consumption per cantonal population size and language region. The annual consumption is exemplified for the following anaesthetics: A) Propofol, B) Rocuronium, C) Fentanyl, D) Sevoflurane. The sub-plots for each anaesthetic present the yearly consumption of anaesthetics per size of the cantonal population (dots), sub-stratified by language region DE–German region (blue), FR–French region (red) and IT–Italian (yellow) region. Further, the fitted linear regression (red line) is displayed with its standard error (turquoise shaded area) and the corresponding linear equation, with *R*^*2*^- and *p*-values are shown on the left upper corner of each sub-plot.

The annual anaesthetic consumption varies regionally, with the German region having a higher annual need of propofol per 100’000 inhabitants than the Italian and French language regions, which have a higher sevoflurane consumption (Fig 2). The annual amount of anaesthetics employed on a cantonal base is strongly associated with the size of the population in the case of propofol, rocuronium and fentanyl (*R*^*2*^ *>0*.*93*; Fig 3), but shows a less stringent correlation in the case of sevoflurane (*R*^*2*^ *= 0*.*78*; Fig 3). These figures root in the different distribution of anaesthesia institutions in each language region and also on a differing general anaesthesia approach, as previously illustrated (Table 1 and Fig 1).

## Discussion

This is the first study that systematically investigates the numbers and types of anaesthesia services in Switzerland. The survey was conducted together with detailed analysis on anaesthetic drug use of a large, representative Swiss anaesthesia index institution and allowed us to make calculations and projections for the annual national need and supply of anaesthetics in Switzerland. This information served as a basis to actively manage the anaesthesia drug supply chain in times of drug shortages due to the COVID-19 pandemic.

Switzerland lies in the heart of Europe and is surrounded by three major language regions–German, French and Italian. We could show significant differences in the number of anaesthesias and anaesthesia time per inhabitants as well as type of anaesthesia provided between the three regions (Table 1). If one wants to apply our data to the surrounding countries, these differences in the various language regions may be taken into account. Switzerland is a wealthy country with a health expenditure around 11.2% of the gross domestic product (GDP, 2018; https://www.bfs.admin.ch/bfs/en/home.html). Regarding staffing, we are privileged by having 2’667 anaesthesiologists (77% board certified, 23% in residency; 2019), i.e. 31.3 for every 100’000 inhabitants [9]. The World Federation of Societies of Anaesthesiologists (WFSA) has released a map showing, country by country, the number of physician anaesthesia providers (anaesthesiologists) worldwide. They found that wealthy countries have around 20 to 30 anaesthesiologists for every 100,000 people; but in sub-Saharan Africa and parts of Asia, there are often less than one for every 100,000 people [10].

Nevertheless, during the first wave of the COVID pandemic, Switzerland as most countries worldwide faced serious drug supply shortages in anaesthesia and the ICU. This was due to the rapid and tremendous increase in orders and consumption of specific anaesthesia and ICU medications. Other factors reinforced this negative effect, such as the economic pressure of the last years created by globalisation, resulting in the centralisation of production at a small number of locations. In addition, lean inventory management and just in time ordering is leading to low stocks at the end of the supply chain with minimal reserves. And finally, the impact of failures and quality problems in the manufacturing chain has become global rather than limited to a specific locality today. In Switzerland, national authorities (Federal Office of Public Health, Federal Office of National Economic Supply) and private initiatives such as drugshortage.ch monitor the drug supply chain and report potential and existing shortages. Despite their activities, the problem of drug shortage hasn’t been solved by far. Thereby, the media and national politics have taken up the theme on multiple occasions and are calling for solutions. In order to improve the current situation and to be prepared for future shortages, further measures have to be taken in addition to those monitoring activities already in place. There needs to be a coordinated collaboration between different national authorities (in Switzerland the federal and cantonal authorities), service providers and the industry, as well as the adoption of a cross-border multi-national approach.

In order to be prepared for any future drug shortages, each country needs to know their interdependencies within the entire supply chain (from manufacture, storage and market access to pricing and remuneration) and work out a catalogue of measures how to best manage and maintain access to all essential medicines. As a basis, the World Health Organization has developed a list (https://www.who.int/medicines/publications/essentialmedicines/en/) of essential medicines and revises it regularly. It focuses on therapeutic necessity. National authorities together with pharmacists play a key role in coordination and keeping up the supply chain [11]. Nevertheless, replenishment but also resources may be limited and each health provider from a single practitioner to large hospitals need their own strategies, how to deal with this upcoming challenge. In addition to increased stockpiling, ways must be found to proactively adjust the use of critical drugs and prepare for the threat, adjusting existing protocols and examining the use of safe alternatives.

The annual rate of anaesthetic procedures in Switzerland is currently 12’445 per 100’000 inhabitants, with some differences in our three language regions (Table 1). This number of anaesthesias, however, may overestimate the true annual rate of anaesthetized patients since some patients may have received several anaesthetic procedures in one year. There are numerous studies on surgical volume in different countries, and this number can serve as a rough approximate for anaesthesia procedures [12]. According to a recent publication, Switzerland had a surgical volume of 11’190 per 100’000 people in 2015, a number comparable to the U.S. and other high-income countries [13]. Specific reports on anaesthesia activity however are sparse. Clergue *et al*. conducted one of the few studies and reported similar anaesthesia activities in France compared to our findings [14, 15]. The authors performed a national survey of anaesthesia services in 1996 and found an annual rate of anaesthetic procedures around 13’500 per 100’000. If anaesthesias for gastrointestinal endoscopies (16% of all French anaesthesia activities in this survey) are excluded, this would lead to a total number of 11’340 anaesthesias per 100’000. A French follow-up study in 2010 showed that the number of anaesthesias has increased significantly over the years (+42.7%) [16, 17]–a trend that goes in parallel to the increase in surgical activity worldwide. In this study, they reported an annual anaesthesia rate of 17’500 (15’050 after deduction of 16% for gastrointestinal endoscopies) per 100’000 in France [16, 17]. Another study from the UK analysed anaesthesia activities in their public National Health Service (NHS) [18]. In 2013, they found significantly lower annual anaesthesia activity of 5’750 per 100’000. For Switzerland, the anaesthesia rate in public hospitals would be 8’065 anaesthetic procedures per 100’000. Those numbers however cannot be compared 1:1 because proportions of anaesthesias done in public, private and office-based settings are different in both countries.

The distribution of different types of anaesthesia is shown in Table 1 and Fig 1. In Switzerland, GA (TIVA, inhalation, combined) is done in 67% of all anaesthesias, compared to 77% in France (24 years ago) [14] and 77% in the UK (7 years ago) [18]. Although these figures are different, they are nevertheless comparable, since one must take into account the different periods of observation as well as the different definitions of types of anaesthesia in the different countries. Regional anaesthesia (RA) is performed in 28% of all anaesthetic procedures in Switzerland (RA 20%, combined GA & RA 8%). The current overall rate of RA in Switzerland is 5% higher comparing these numbers to the anaesthesia survey in France from 1996 [14, 15]. Looking at the epidemiology and trends however, RA techniques (especially peripheral nerve blocks) are increasingly being used, not least because of the emerging ultrasound technology [19, 20]. So we assume that today, the rate of RA in France is definitively higher and thereby comparable to Switzerland.

The current study has some limitations. *First*, the results of the survey are specific to Switzerland and can be applied to other countries to a limited extent only. We have broken down our figures according to our three different language regions and types of anaesthesia institutions. This might help when comparing our data to neighbouring countries. However, every country has its own characteristics and definitions of anaesthesia including type of anaesthesia. In Switzerland for example, most sedations in gastroenterology, pulmonology, cardiology and interventional radiology are done by non-anaesthetist and are therefore not included in the number of reported annual anaesthesias. *Second*, we had to rely on a reference institution to estimate the Swiss-wide annual consumption of anaesthetics. The reason for this is that there are no data available on drug consumption at the individual anaesthesia departments in Switzerland. Accordingly, we used available data from a well-documented department and performed our projections with certain assumptions. Of course, there may be uncertainties in the extrapolations, as this department also has its peculiarities and may deviate from the Swiss standard. *Third*, in our projections, we focused on anaesthetics that are either being used very frequently or that have been classified critical with regard to their supply by the pharmacy of the index institution or by the Swiss Federal Office of Public Health (Table 2). Furthermore, we only looked at anaesthetics given during the anaesthesia period. Thereby, no calculations were made for drugs used in pain therapy following anaesthesia in the recovery room and the first days after surgery. *Last*, as mentioned earlier, other specialists require these critical anaesthetic drugs. Particularly worthy of mention is the intensive care unit, non-anaesthetists sedations as mentioned above and the emergency medical services. In addition, analgesics and sedatives are required in pain medicine, addiction and palliative care.

## Conclusions

Drug shortages are a serious problem that has increased in recent years. Due to the unexpected occurrence of the COVID-19 pandemic, such a shortage has suddenly appeared in Swiss anaesthesia practice on a previously unknown scale. Besides shortfalls of overseas manufacturing, a misallocation of medications within the nation’s internal supply chains is also of major concern. Therefore, active management, transparent information, and timely communication are essential to ensure a steady supply chain of key therapeutic medications, especially during a pandemic. It is imperative to have a detailed understanding of the number and types of anaesthesias. As long as these information are not available, published surgical volume [13] combined with our data on selected drugs for anesthesia services (Table 2) can be used as a starting point to estimate the national anaesthetic need of a given country. This knowledge forms the basis for any drug supply chain management in anaesthesia.

## Supporting information

S1 File Questionnaire. (DOCX)

S1 Table Average use of most relevant drugs in 2019 per one anaesthesia delivered at index anaesthesia institution. (DOCX)
