# Supplementary material for: Analysis of anaesthesia services to calculate national need and supply of anaesthetics in Switzerland during the COVID-19 pandemic
Source: PLoS One. 2021 Mar 19;16(3):e0248997. doi: 10.1371/journal.pone.0248997 (PMC7978279; doi:10.1371/journal.pone.0248997)
Supplement: S1 File — (DOCX) [file pone.0248997.s001.docx]

##
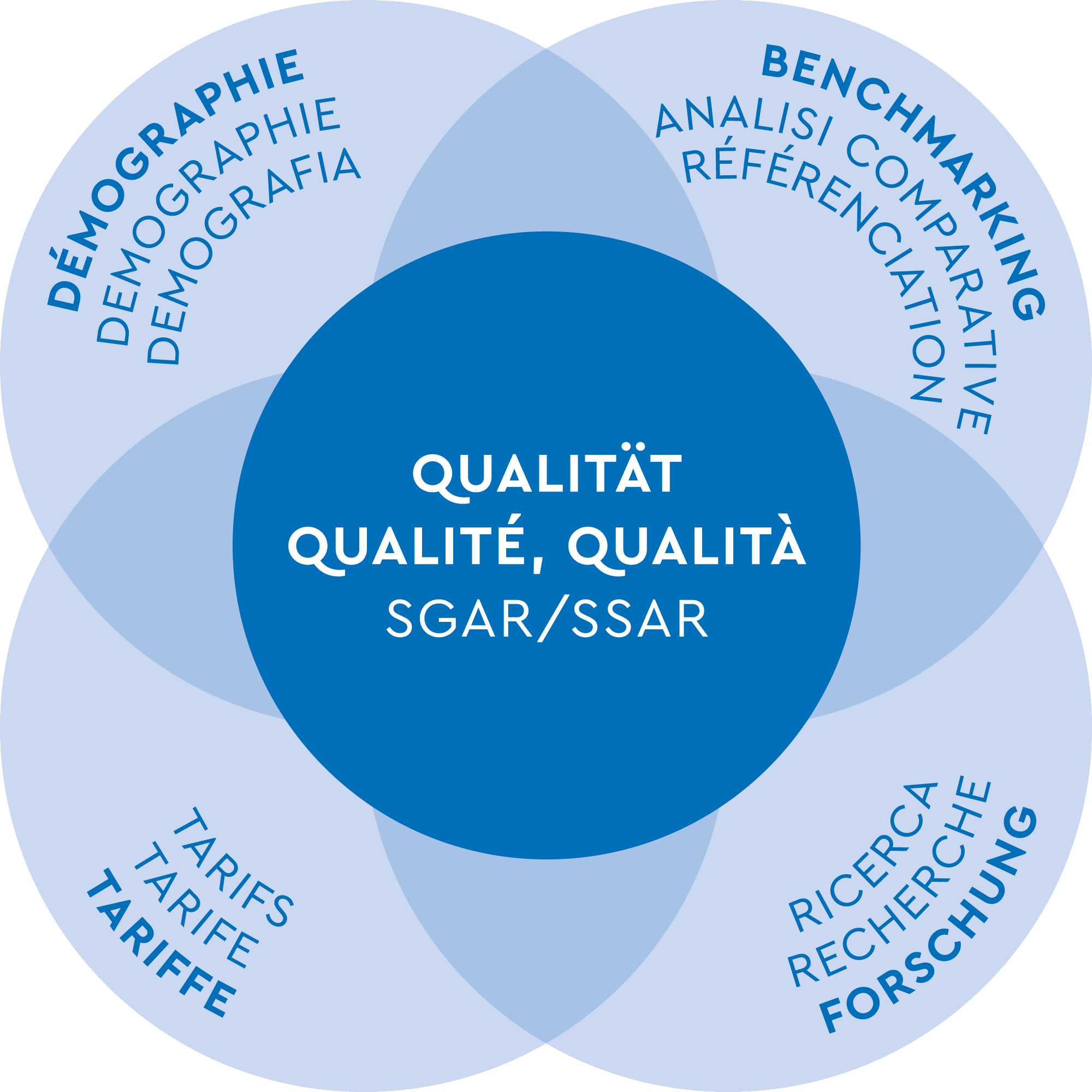
S1 File. Questionnaire.

**
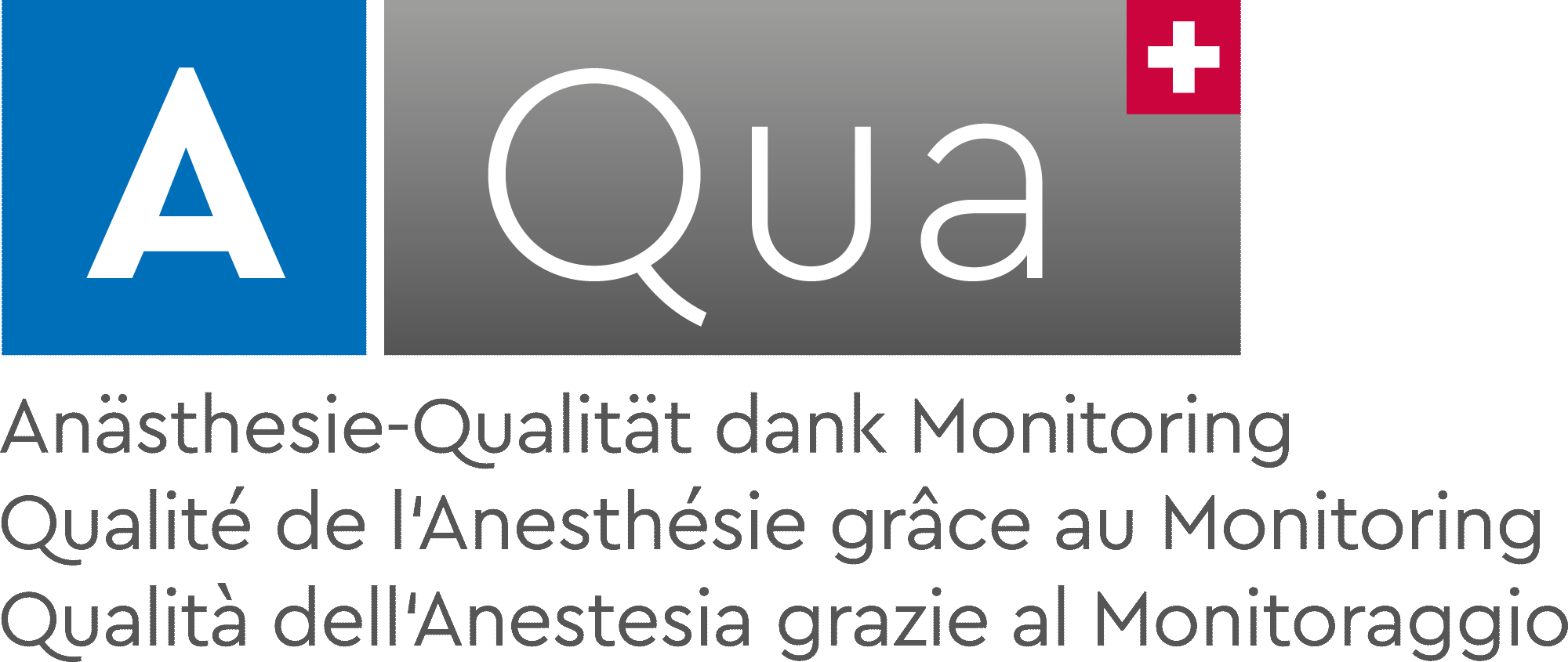
**

**SSAR survey in Swiss anaesthesia institutions due to Swiss Federal Office of Public Health (BAG) request to coordinate the supply of anesthetics in the context of the COVID-19 pandemic**

| **Your anaesthesia department** (= Institute, department, practice, company for anaesthesia/anesthesiology etc.) | | |
| --- | --- | --- |
| **Name of the anaesthesia department** with associated hospital (if available) | xx | |
| **Last and first name, head of department** | xx | |
| **E-mail address, head of department** | xx | |
| **Your anaesthesia services per year** year of reference: 2019 | | |
| **Number of anaesthesias**  (total number of all anaesthesias of the department/institution, 2019) | | xx |
| **Total anaesthesia time**  (Sum of all "anaesthesia care times = start AQ1 to end AQ12 of anaesthesia care" in hours, 2019) | | xx |
| **Type of anaesthesia**  (indicated in % share; control: the sum of all % shares of the 5 lines below must equal 100%!) | | |
| **General anaesthesia, TIVA**  [total intravenous anaesthesia (no inhalation); this type of anaesthesia also includes deep sedation / analgosedation, resuscitation and anaesthesia patient transport within the hospital] | | xx |
| **General anaesthesia, by inhalation**  [inhalational anaesthesia (also with i.v. induction) and mixed anaesthesia (inhalational and intravenous)] | | xx |
| **Regional anaesthesia**  [pure regional anaesthesia (with and without sedation, spontaneous breathing maintained, no respiratory aid)] | | xx |
| **Combination anaesthesia: general + regional anaesthesia**  [planned combined anaesthesia: regional anaesthesia and purely intravenous general anaesthesia or general anaesthesia with inhalants] | | xx |
| **MAC (Monitored Anaesthesia Care)**  [according to Def. TARMED; MAC with and without analgosedation]. | | xx |
