# Supplementary material for: Analysis of anaesthesia services to calculate national need and supply of anaesthetics in Switzerland during the COVID-19 pandemic
Source: PLoS One. 2021 Mar 19;16(3):e0248997. doi: 10.1371/journal.pone.0248997 (PMC7978279; doi:10.1371/journal.pone.0248997)
Supplement: S1 Table — (DOCX) [file pone.0248997.s002.docx]

## S1 Table. Average use of most relevant drugs in 2019 per one anaesthesia delivered at index anaesthesia institution.

| *Selected* drugs used per general anaesthesia (GA) delivered |  |
| --- | --- |
| Hypnotics |  |
| *GA, TIVA*   - Propofol 1% (induction and maintenance)   *GA, inhalation*   - Propofol 1% 200 mg or Thiopental 0.5 g or Etomidate 20 mg (induction) - Volatile anaesthetic drug (Sevoflurane or Desflurane; maintenance) ^c^ | 1'102 mg  1 vial  35 mL |
| Opioids |  |
| - Fentanyl - Remifentanil - Morphin | 584 mcg  452 mcg  6 mg |
| Muscle relaxants |  |
| - Rocuronium ^a^ - Reversal mandatory (neostigmine 2.5 mg or sugammadex 200 mg) ^b^ | 54 mg  37% |
| Anti-emetics |  |
| - Dexamethasone - Ondansetrone | 2.4 mg  1.5 mg |

^a^ mean dose of rocuronium used for patients under general anaesthesia with endotracheal intubation was 90 mg, 61% of these patients were reversed

^b^ standard reversal is either 1 vial of neostigmine 2.5 mg plus gycopyrrolate 0.5 mg (92%) or sugammadex 200 mg (8%)

^c^ Sevoflurane is predominantly being used at index institution (92%)

| *Selected* drugs (during anaesthesia only, no post-op) used per regional anaesthesia (RA alone or combined with GA) delivered |  |
| --- | --- |
| Local anesthetics |  |
| *Spinal anaesthesia ^c^*   - Bupivacain 0.5% hyperbaric   *Epidural anaesthesia ^c^*   - Lidocaine 2% (test dose, top up epidural) - Ropivacaine 0.2% (running epidural)   *Peripheral nerve block (incl. trunc) ^c^*   - Mepivacaine 1% - Ropivacaine 0.5% | 20 mg  174 mg  102 mg  380 mg  38 mg |

^c^ RA – at index institution 32% spinal, 30% epidural, 38% peripheral and others

| *Selected* drugs used per every anaesthesia delivered |  |
| --- | --- |
| Cardiovascular drugs |  |
| - Ephedrine - Norepinephrine - Phenylephrine - Atropine - Epinephrine - Clonidine | 32 mg  447 mcg  41 mcg  73 mcg  40 mcg  16 mcg |
| Others |  |
| - Crystalloids (lactated Ringer, Ringerfundin, NaCl 0.9%) - Carrier solutions (NaCl 0.9%, D5W) - Midazolam | 1'362 mL  187 mL  0.3 mg |

The above lists are not conclusive. Only those drugs have been listed which are either used very frequently or which have been classified as critical by the pharmacy of the index institution with regard to their supply.
